# The phantom nail: ligamentum arteriosum calcification in trauma

**DOI:** 10.36416/1806-3756/e20260128

**Published:** 2026-08-13

**Authors:** Huiyan Luo, Weiwei Liao

**Affiliations:** 1. Medical Imaging Center, Ganzhou Hospital-Nanfang Hospital, Southern Medical University (Ganzhou People’s Hospital), Ganzhou, Jiangxi, China.

A two-year-old boy presented two hours after a nail gun injury. He was irritable but lacked respiratory distress or hemoptysis. Physical examination revealed a 3-mm wound at the right parasternal 5th intercostal space. Chest CT identified a 5 × 9 × 5 mm high-density nodular opacity in the left aortopulmonary window (Figures 1A and 1B). Surrounding tissue planes were clear, and no pneumothorax or effusion was present. A retained metallic foreign body was tentatively diagnosed.

**Figure 1:**
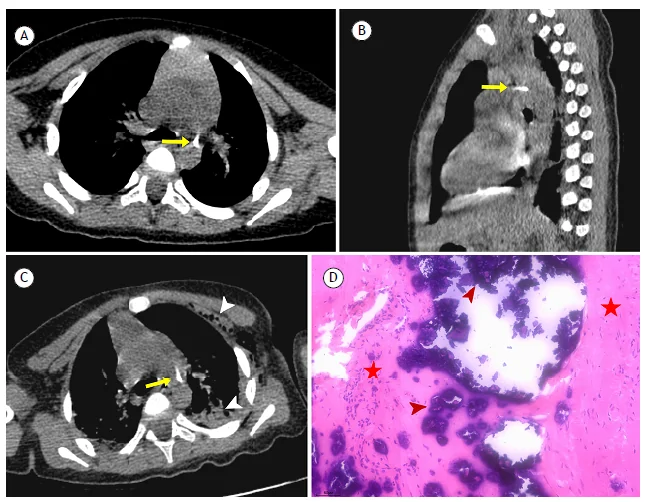
Imaging and histopathological findings of a two-year-old boy. Axial (A) and sagittal (B) non-contrast CT images show a high-density nodular image (arrows) within the left aortopulmonary window, initially suspected as a metallic foreign body. Repeat CT following the first thoracotomy (C) reveals the persistence of the high-density finding (arrow), along with postoperative soft tissue emphysema and pulmonary infiltrates (arrowheads). Histopathology (D) demonstrates abundant collagenous fibrous tissue (stars) and irregular calcifications (arrowheads), confirming *ligamentum arteriosum* calcification. (H&E; magnification, ×200).

An emergency thoracotomy was performed; however, exploration revealed no hematoma, perforation, or foreign body within the pleura, mediastinum, or thoracic cavity. Intraoperative fluoroscopy was also negative. An emergent postoperative CT confirmed the persistence of the high-density image (Figure 1C). Although a calcified focus was suspected at this point, a second surgery was performed due to strong parental insistence. Firm tissue was palpated at the *ligamentum arteriosum*, and biopsy confirmed *ligamentum arteriosum* calcification (LAC) (Figure 1D).

LAC is a common pediatric imaging pitfall, often misidentified as an esophageal-perforating foreign body in cases of ingestion.^1^ ^,^ ^2^ Unlike those reports, this misleading projectile trauma history resulted in misidentifying LAC as a penetrating foreign body. Diagnostic uncertainty necessitated two surgeries to satisfy the family’s concerns. Recognizing LAC-especially without secondary traumatic changes like pneumothorax or mediastinal hematoma-is the ultimate safeguard against unnecessary intervention in this vulnerable population.

## References

[1] Kakiuchi T, Nakayama A (2020). Calcification of the ligamentum arteriosum with suspected fish-bone foreign body on computed tomography. J Gen Fam Med.

[2] Chu XY, Cui Y, Gao Z (2020). Ligamentum arteriosum calcification that presented as an esophageal perforation caused by duck bone ingestion. J Int Med Res.

